# New Routes in GPCR/β-Arrestin-Driven Signaling in Cancer Progression and Metastasis

**DOI:** 10.3389/fphar.2019.00114

**Published:** 2019-02-19

**Authors:** Anna Bagnato, Laura Rosanò

**Affiliations:** Unit of Preclinical Models and New Therapeutic Agents, IRCCS-Regina Elena National Cancer Institute, Rome, Italy

**Keywords:** cancer, β-arrestin, G protein-coupled receptors, cytoskeleton, motility

## Abstract

Tumor cells acquire invasive and metastatic behavior by sensing changes in the localization and activation of signaling pathways, which in turn determine changes in actin cytoskeleton. The core-scaffold machinery associated to β-arrestin (β-arr) is a key mechanism of G-protein coupled receptors (GPCR) to achieve spatiotemporal specificity of different signaling complexes driving cancer progression. Within different cellular contexts, the scaffold proteins β-arr1 or β-arr2 may now be considered organizers of protein interaction networks involved in tumor development and metastatic dissemination. Studies have uncovered the importance of the β-arr engagement with a growing number of receptors, signaling molecules, cytoskeleton regulators, epigenetic modifiers, and transcription factors in GPCR-driven tumor promoting pathways. In many of these molecular complexes, β-arrs might provide a physical link to active dynamic cytoskeleton, permitting cancer cells to adapt and modify the tumor microenvironment to promote the metastatic spread. Given the complexity and the multidirectional β-arr-driven signaling in cancer cells, therapeutic targeting of specific GPCR/β-arr molecular mechanisms is an important avenue to explore when considering future new therapeutic options. The focus of this review is to integrate the most recent developments and exciting findings of how highly connected components of β-arr-guided molecular connections to other pathways allow precise control over multiple signaling pathways in tumor progression, revealing ways of therapeutically targeting the convergent signals in patients.

## Introduction

G protein-coupled receptors (GPCRs) constitute the largest family among the membrane proteins, playing an important role not only in mediating physiological function but also controlling the recruitment and activation of intracellular molecules associated with human diseases, including cancer (Lagerstrom and Schioth, 2008; Hauser et al., 2017; Insel et al., 2018). Agonist-activated GPCRs couple to heterotrimeric G proteins, thus facilitating exchange of GDP by GTP in the Gα subunits followed by dissociation from the βγ dimers and transiently interacting with specific effectors to trigger canonical signal transduction cascades. The phosphorylation of GPCRs by G protein-coupled receptor kinases (GRKs), a subfamily of AGC (protein kinase A/G/C-like) kinases originally identified as inhibitors of GPCR signaling, promotes the recruitment to the phosphorylated receptor of the cytosolic proteins β-arrestins (β-arrs), leading to GPCR desensitization (Gurevich et al., 2012).

In the classical view, GPCR signaling is mediated by G proteins, while β-arr recruitment is associated with signaling desensitization of GPCR. In this old view, the competition between G proteins and β-arrs, β-arr1 or β-arr2, for an activated GPCR determines the signal termination, hampering further G protein signaling (Luttrell and Lefkowitz, 2002; Sente et al., 2018). Also, β-arrs promote clathrin-dependent-endocytosis of activated GPCR (Luttrell and Lefkowitz, 2002; Sente et al., 2018). One of the most important breakthroughs in GPCR field during the past decades was that the signal transduction of GPCRs is also strictly linked to β-arr. Beyond their known roles in GPCR desensitization/internalization, β-arrs have been implicated in the control of multiple outcomes, acting as multifunctional scaffold proteins and signaling transducers, crucial for intracellular signal propagation and amplification, and governing different cellular effects (Miller and Lefkowitz, 2001; DeFea, 2008; Shukla et al., 2011; Peterson and Luttrell, 2017). Almost two decades after the first evidence, GPCR field now encompasses an extensive knowledge that β-arrs integrate signals arising from GPCR with intrinsic cellular pathways in human disease, initiating waves of intracellular signaling in a G protein-independent manner and allowing the discovery of new therapies targeting selectively β-arr-mediated circuits, known as biased arrestin-biased agonism (Smith et al., 2018). Moreover, since β-arr-biased signaling requires phosphorylation of GPCRs by GRKs to promote high-affinity binding of β-arr to GPCRs and GRK subtypes might have preferential phosphorylation and trigger unique conformational changes in GPCRs, studies of β-arr-biased signaling might consider also the involvement of GRKs in cancer-related signaling pathways (Heitzler et al., 2012). In this regard, different isoforms of GRKs are able to modulate the response to many GPCRs involved in tumoral signaling *via* its direct interaction with other components of transduction cascades, as well outlined in a recent review (Nogués et al., 2018). Therefore, GRKs would also be considered critical to control the fate of β-arr-dependent signaling of GPCRs and as potential therapeutic targets in cancer.

Recent pharmacological studies on the paradigm of biased agonists, where a particular biased ligand can generate a GPCR conformation able to lead to a distinct functional outcome, usually either G-protein or β-arr-dependent signaling but not both, suggest that current GPCR-based therapeutics could be improved by increasing anticancer efficacy (Smith et al., 2018). Moreover, computational and atomic level dynamic simulation approaches provided new details linking phosphorylation of GPCR, β-arr interactions, and β-arr-dependent signaling, supporting the “barcode hypothesis,” in which distinct patterns of GPCR phosphorylation trigger specific conformational states of β-arr with specific functional outcomes (Srivastava et al., 2015). In addition, remarkable advances in the GPCR structural biology field deeply demonstrated that specific ligands, by stabilizing particular sets of conformations and permitting the interaction with specific effectors, might achieve specific efficacies for selected signaling pathway (Rosenbaum et al., 2009). Recently, this conceptual framework has been refined, whereby the activated GPCR might lead the formation of a “supercomplex,” where GPCR and β-arr1 form a unique signaling module with G-protein (Marshall, 2016; Thomsen et al., 2016). These findings support the hypothesis of a new way to signal, by concomitant binding of G proteins and β-arr to activated receptors, further providing an additional paradigm in GPCR-driven signaling transduction.

## β-Arrestins as Scaffold Proteins in GPCR Signaling

**Figure 1:**
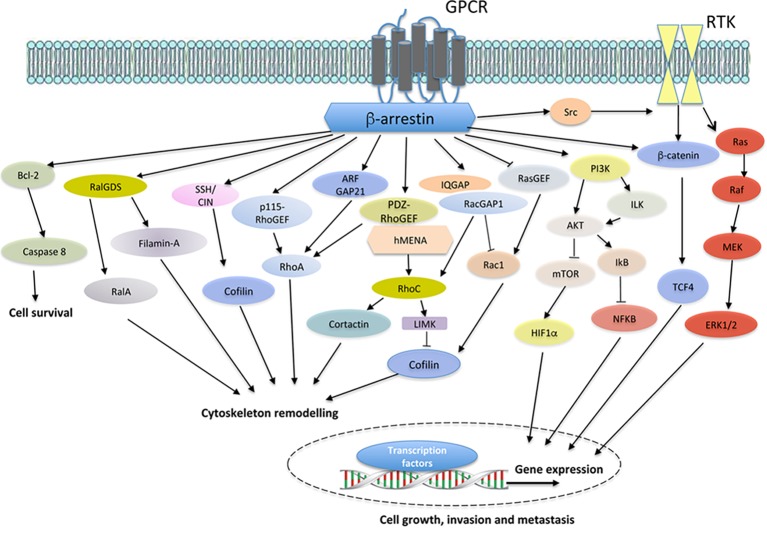
Model of GPCR/β-arr-dependent signal pathways controlling cell survival, cytoskeleton remodeling, and gene expression, leading to enhanced cell growth, invasion and metastasis. In different cancer cells, the binding of ligands to cognate GPCRs leads to the recruitment of β-arrestin (β-arr), which might activate diverse signal-transduction pathways, including Bcl2 and downstream caspase 8. The crosstalk with receptor tyrosine kinases (RTKs), through the recruitment and activation of Src, results in downstream pathway activation, such as members of the Ras/Raf/MEK/ERK family and β-catenin/TCF4. Moreover, GPCR stimulation activates PI3K, leading to AKT/integrin-linked kinase (ILK) signaling and mTOR inhibition. Beyond the cytosolic functions, β-arr might regulate hypoxia-inducible factor 1α (HIF1α) at the levels of transcription, leading to nuclear entry and binding to hypoxia-response elements and the gene transcription. Similarly, β-arr might activate nuclear factor-κB (NF-κB) signaling *via* inhibition of NF-κB inhibitor (IκB), resulting in the dissociation and subsequent nuclear localization of active NF-κB. At the same time, the interaction of β-arr with actin regulators, such as Filamin-A and LIMK, and ser/thr phosphatases, such as SSH and CIN, leads to enhanced cofilin activity in actin cytoskeleton effects. In addition, GPCR activation might promote the interaction between β-arr and either RHO-guanine nucleotide exchange factors (RHO-GEFs), such as p115RhoGEF or PDZ-RhoGEF, or Rho GTPase-activating proteins (RhoGAPs), such as ARF-GAP21, to activate RhoA GTPase and regulate actin remodeling. β-arr can also bind RalGDS to activate RalA GTPase in cytoskeletal reorganization. Moreover, the interaction of β-arr1 with PDZ-RhoGEF and members of ENA/VASP family, hMENA, might lead to RhoC GTPase activation, causing LIMK-dependent cofilin inhibition and cortactin activation, enhancing invasive behavior. At the same time, β-arr might bind IQGAP1 and RacGAP1, leading to the suppression of Rac1 activity and favoring activation of RhoC and invadopodia functions. The inhibition of β-arr-dependent RASGFR2 activates Rac1 promoting actin polymerization through cofilin activity. β-arr acts as hub regulating several cellular processes related to cancer progression *via* its interaction with different components of transduction cascades.

In cancer cells and in a cell context- and cancer type-dependent manner, the pools of β-arr-dependent multiprotein complexes can be found localized to different intracellular compartments, as bound to the cytoskeleton, as endocytic adapters acting on specific signalosomes in endosomes and interacting with signaling proteins involved in gene transcription, protein ubiquitination, and cytoskeletal remodeling, among others (Ma and Pei, 2007; Sobolesky and Moussa, 2013; McGovern and DeFea, 2014; Black et al., 2016; Jean-Charles et al., 2016; Rosanò and Bagnato, 2016; Chaturvedi et al., 2018; Eichel and von Zastrow, 2018; Song et al., 2018). β-arr-dependent multiprotein complexes, transducing the GPCR signals, regulate the functionality of different tyrosine kinase receptor family members and directly control cytosolic, cytoskeletal remodeling or nuclear signaling components of pathways relevant for tumor growth, invasiveness, and metastatic progression (Figure 1). Through these functions, both β-arrs foster a plethora of signaling pathways, including members of the mitogen-activated protein kinase (MAPK), AKT, PI3K, Wnt, Hedgehog, E3 ubiquitin ligases, PTEN, nuclear factor-kB, and regulators of small GTPase activity. To expand the intracellular communication, agonists of GPCRs can activate tyrosine kinase receptors (RTK), through a signal cross talk. This can occur *via* a mechanism by a GPCR-mediated activation of proteases operating the ectodomain shedding of a membrane bound pro-ligand, such as heparin-binding epidermal growth factor (Hb-EGF), or by the intercellular activity of GPCR-activated tyrosine kinase, completely independent of ligand binding (Rosanò and Bagnato, 2016; Crudden et al., 2018). Moreover, accumulating evidence recognizes that the transactivation of RTKs by GPCRs is not unidirectional, as the cross talk between RTKs and GPCRs is reciprocal, GPCRs can be activated by RTKs, and β-arr can be used by RTKs, as in the case of insulin-like growth factor type 1 receptor (Girnita et al., 2005, 2007; Zheng et al., 2012; Crudden et al., 2018) or platelet-derived growth factor receptors (Pyne and Pyne, 2017). In both mechanisms, it is well known that some GPCRs use β-arr to execute and transduce this cross talk between GPCRs and RTKs, governing multiple cellular processes in cancer invasion and metastasis. Proteomic studies in cancer cells demonstrated a very impressive diversity of signaling cascade molecules, which can be engaged by β-arrs for a positive or negative signaling regulation (Xiao et al., 2007; Parisis et al., 2013; Xiao and Sun, 2018), underscoring the importance of GPCR-driven β-arrs in shaping and fine-tuning signaling in cancer progression.

β-arrs are expressed in human tumors and mediate multiprotein signaling complexes, in which β-arr acts as membrane, cytosolic, or nuclear scaffold and signal transducer, culminating in multifaceted signaling processes, such as cell growth and proliferation, drug resistance, cell migration, invasion, and metastasis (Ma and Pei, 2007; Sobolesky and Moussa, 2013; Black et al., 2016; Jean-Charles et al., 2016; Rosanò and Bagnato, 2016; Chaturvedi et al., 2018; Song et al., 2018) (Table 1). In this review, we summarize new specific routes of β-arr-mediated signaling of GPCR in cancer, focusing on invasive behavior, sustained by complex machinery that includes physical interaction with adaptor proteins.

**Table 1:** β-arrestins in cancer.

Tumor	β-arr	Role in tumor progression and metastasis	References
Ovarian cancer	β-arr1/β-arr2	Chemoresistance, angiogenesis, invadopodia, invasion, EMT, metastasis	Rosanò et al., 2009, 2012, 2014; Cianfrocca et al., 2010, 2014, 2016; Semprucci et al., 2016; Chellini et al., 2018; Di Modugno et al., 2018
Lung cancer	β-arr1/β-arr2	EMT, invasion, chemoresistance	Wang et al., 2018; Tsai et al., 2019
Prostate cancer	β-arr1/β-arr2	Cell growth, migration, invasion, EMT, angiogenesis, metastasis	Ma et al., 2014; Zecchini et al., 2014; Purayil et al., 2015; Duan et al., 2016; Kong et al., 2018
Acute lymphoblastic leukemia	β-arr1	Cell propagation and senescence	Qin et al., 2014; Liu et al., 2017; Ferrandino et al., 2018
Chronic myeloid leukaemia	β-arr1/β-arr2	Tumor cells initiation and growth, stemness	Fereshteh et al., 2012; Qin et al., 2014
Colorectal cancer	β-arr1/β-arr2	Cell proliferation, apoptosis, chemoresistance, migration, invasion and metastasis	Buchanan et al., 2006; Jin et al., 2013; Cianfrocca et al., 2017; Ren et al., 2018
Gastric cancer	β-arr1	Cell proliferation	Alvarez et al., 2009
Osteosarcoma/ Ewing’s sarcoma	β-arr1	Cell sensitivity, proliferation and invasion	Zheng et al., 2012; Zhang et al., 2014
Breast cancer	β-arr1/β-arr2	Cell proliferation, apoptosis, chemotaxis, invasion, invadopodia, metastasis, angiogenesis, multidrug resistance	Sun et al., 2002; Ge et al., 2004; Li et al., 2009; Zhao et al., 2009; Zajac et al., 2011; Shenoy et al., 2012; Alemayehu et al., 2013; Jing et al., 2015; Goertzen et al., 2016
Medulloblastoma	β-arr1/β-arr2	Cancer stem cells self-renewal	Miele et al., 2017; Infante et al., 2018
Renal cancer	β-arr2	Cell growth, metastasis	Masannat et al., 2018
Melanoma	β-arr1	Cell migration, vasculogenic mimicry	Spinella et al., 2013
Pancreatic cancer	β-arr2	Cell proliferation and invasion	Heinrich et al., 2012

## β-arr1 as Scaffold for Cytoskeleton Remodeling in Tumor Cell Motility

Cell motility is a complex process in which cells change shape following activation of signaling pathways that control cytoskeleton dynamics and the turnover of cell-matrix and cell-cell junctions (Friedl and Alexander, 2011; Lambert et al., 2017). The primary driving force guiding cell motility is linked to actin assembly/disassembly within cells, in which different proteins might regulate the major steps in actin remodeling, as activation of proteins breaking the existing filaments into smaller fragments to create free barbed ends and protein nucleators facilitating association of actin monomers into filaments and membrane protrusions. Some of these processes depend on environmental cues, including ligand-dependent activation of GPCR, and their spatial/temporal regulation is mediated by proteins interacting directly or indirectly with the actin and microtubule cytoskeleton elements, in proximity with signaling proteins. During tumor cell migration, chemotaxis, and metastasis, β-arrs mediate GPCR-driven effects on actin cytoskeleton remodeling by orchestrating activation and localization of selected proteins at the leading edge and generating the necessary forces for movement (Zoudilova et al., 2007, 2010; Min and Defea, 2011; Ma et al., 2014). Although the first evidence showed that β-arr-MAPK complexes control actin cytoskeletal reorganization at the leading edge during cell migration (Sun et al., 2002; Ge et al., 2004; Décaillot et al., 2011), many other studies highlighted that β-arrs function as master signalosome scaffold for specific cytoskeleton-related signaling molecules, including c-Src, filamin, cofilin, and small monomeric GTPases, to connect GPCRs to the cytoskeleton and cell shape changes (Bhattacharya et al., 2002; Barnes et al., 2005; Hunton et al., 2005; Buchanan et al., 2006; Scott et al., 2006; Zoudilova et al., 2007, 2010; Li et al., 2009; Rosanò et al., 2009; Godin et al., 2010; Min and Defea, 2011; Ma et al., 2012; Semprucci et al., 2016; Shishkin et al., 2016; Tocci et al., 2016). Cofilin is considered as one of the primary actin filament severing proteins, operating a rapidly disassembling of existing filaments and promoting generation of actin filament extension and the formation of the leading edge during chemotaxis (Shishkin et al., 2016). The function of β-arr, by interacting with cofilin and phosphatases/proteases or by mediating the activity of small GTPases, is a prerequisite for controlling phosphorylation/inactivation-dependent cofilin activity (Zoudilova et al., 2007, 2010; Min and Defea, 2011). Growing evidence demonstrated that β-arr regulates the activity of RhoA, in the family of Rho GTPases, by interacting with guanine nucleotide exchange factors (GEFs), such as p115RhoGEF and PDZ-RhoGEF, or GTPase activating protein (GAPs), such as ARFGAP21, in regulating stress fiber assembly/disassembly, or with Ral-GDS to regulate RalA for controlling membrane ruffling and cell migration, as observed for activation of cognate receptors for angiotensin AT1A (ATII1A), beta-2 adrenergic (β2AR), formyl-Met-Leu-Phe, lysophosphatidic acid (LPA), or endothelin-1 (ET-1) (Bhattacharya et al., 2002; Barnes et al., 2005; Hunton et al., 2005; Li et al., 2009; Godin et al., 2010; Ma et al., 2012; Semprucci et al., 2016; Tocci et al., 2016). Other studies showed that GPCR-dependent cytoskeletal rearrangement and membrane protrusion formation might depend on the interaction of β-arr with the actin-binding protein filamin A (Scott et al., 2006).

The dissemination of cancer cells from primary tumors and their seeding in the metastatic niche often involves the local movement of tumor cells and their penetration into surrounding tissue, which require dramatic reorganization of the cell cytoskeleton and remodeling of extracellular matrix (ECM) (Friedl and Alexander, 2011; Eddy et al., 2017; Lambert et al., 2017; Mrkonjic et al., 2017; Paterson and Courtneidge, 2018). Therefore, invading cells adopt mesenchymal, elongated morphology with focalized proteolytic activities toward ECM, by forming invadopodia (Eddy et al., 2017; Mrkonjic et al., 2017; Paterson and Courtneidge, 2018). In this context, it has been demonstrated that β-arr-related molecular complexes might govern invasive and metastatic behavior by promoting invadopodia formation. The activation of GPCRs, such as ET-1R, initiates downstream signaling cascade and formation of protein complexes mediating actin rearrangement leading to invadopodia formation and ECM degradation, where β-arr1 regulates the spatial distribution of actin cytoskeleton and actin regulators (Bagnato and Rosanò, 2016; Semprucci et al., 2016). This cascade, starting with the interaction of β-arr1 with PDZ-RhoGEF, controlling the spatial distribution of RhoC GTPase and cofilin pathway, represents a critical route by which the tumor cells form active invadopodia, and acquires maximally proinvasive capabilities (Semprucci et al., 2016). Following studies demonstrated that β-arr1-associated molecular complexes during invasive protrusions involve members of the ENA/VASP family, known to regulate the actin-based motility of various cell types, and in particular, hMENA and the invasive isoform hMENAΔv6 (Krause et al., 2003; Gertler and Condeelis, 2011; Di Modugno et al., 2012, 2018). Inputs derived from ET-1R promote the formation of a signaling platform containing β-arr1-hMENA/hMENAΔv6/PDZ-RhoGEF converging on RhoC pathway, favoring pericellular matrix degradation, and conferring also a fitness advantage to tumor cells to breach the endothelial barrier and start the transendothelial migration process (Di Modugno et al., 2018). More recently, a deep understanding of how protein complexes are assembled into a functional unit to enhance specific signaling pathways revealed a new mechanistic link between β-arr1 and the integrin-related protein IQ-domain GTPase-activating protein 1 (IQGAP1), downstream of ET-1R signaling, in shaping cytoskeleton remodeling and invadopodia-dependent ECM degradation (Chellini et al., 2018). Specifically, IQGAP1/β-arr1 acts as small GTPase scaffolding platform, as RacGAP1, to promote Rac1 inhibition and concomitant RhoA,C activation, suggesting that ET-1R-guided β-arr1 interactions determine the convergence and activation/inhibition of specific signals for invadopodia, such as Rho GTPases, where IQGAP1 helps to define the discrete locations and/or time (Chellini et al., 2018). In line with these findings, the activation of the GPCR kisspeptin receptor promotes invadopodia formation in human breast cancer cells *via* β-arr2/ERK (Goertzen et al., 2016). Concordantly, new findings demonstrated a functional link between the tumor suppressor PTEN, scaffolding function of β-arr1, ARHGAP21 and cytoskeletal rearrangements, in driving evolution of 3D morphology phenotypes mimicking colorectal cancer in early step of metastatization (Jagan et al., 2013; Javadi et al., 2017). These new findings disclose so far an unexpected role of β-arr capable to rewire the GPCR signaling networks and activate specific machinery for changing shape, generating invasive protrusions, and remodeling ECM in invasive and metastatic cancer cells, and update the signaling paradigm that targeting GPCR/β-arr1 pathways can represent a possible route of therapeutic intervention (Rosanò et al., 2013; Bagnato and Rosanò, 2016; Goertzen et al., 2016; Semprucci et al., 2016; Chellini et al., 2018; Di Modugno et al., 2018).

## Nuclear function of β-arr1 in Cancer

Among the non-canonical functions of β-arr, many studies demonstrated that nuclear β-arr1 might generate coordinated transcriptional responses to environmental changes, uncovering additional functions of β-arr1 in tumor progression (Kang et al., 2005; Shi et al., 2007; Hoeppner et al., 2012; Yang et al., 2015). To dissect a genomic landscape of β-arr1 in cancer and find direct transcriptional targets, an integrated whole-genome ChIP-Seq analysis and gene expression profiling have been performed in prostate cancer cells exposed to pseudohypoxia, a condition that mimics hypoxia that is frequently encountered within solid tumors. The results of this study revealed a partial overlap between β-arr1 and p300 acetyltransferase-binding sites in the same gene-proximal regions and the presence of non-overlapping sites, suggesting a double-hedged sword of β-arr1 in modulating gene, dependently or independently of p300 (Zecchini et al., 2014). A functional analysis of β-arr1 transcriptome also revealed an enrichment of genes involved in cellular metabolism and the cell cycle, with an overlap with the hypoxia-induced factor-1α (HIF-1α) transcriptome, including known HIF1α target genes involved in angiogenesis and aerobic glycolysis (Shenoy et al., 2012; Zecchini et al., 2014). Concordantly, in ovarian cancer, the activation of ET-1R, by mimicking hypoxia, promotes the interaction between β-arr1/p300 and HIF-1α, enhancing the transcription of genes, such as ET-1 and VEGF, required for tumor cell invasion and proangiogenic effects, operating a self-amplifying HIF-1α-mediated transcription of genes that sustain metastatic process (Cianfrocca et al., 2016). The findings further supporting the nuclear role of β-arr1/p300 in maintaining a more aggressive phenotype demonstrated the interplay with Wnt/β-catenin signaling (Chen et al., 2001; Bryja et al., 2007; Bonnans et al., 2012; Rosanò et al., 2013, 2014; Duan et al., 2016). Downstream of ET-1R activation, β-arr1/p300/β-catenin pathway, also represents a novel bypass mechanism through which this receptor is linked to chemoresistance, cancer stem cells like phenotype, and metastatic behavior (Rosanò et al., 2014). In both androgen-dependent and castration-resistant prostate cancer cells, β-arr1 enhances the binding of androgen receptor (AR) to androgen response elements, favoring cell proliferation, growth, and invasion, as well as *in vivo* tumor formation, local invasion, and distant metastasis (Purayil et al., 2015). Moreover, nuclear β-arr1 suppresses RasGRF2 gene expression through promoter hypermethylation, with consequent controlling of Rac1/cofilin pathways (Ma et al., 2014). An important interplay between nuclear β-arr1 and E2F transcription factor has been demonstrated in non-small cell lung cancers, contributing to the growth and progression of this tumor (Dasgupta et al., 2011; Perumal et al., 2014; Pillai et al., 2015). In myeloid leukemia where β-arr mediates the initiation and maintenance of tumor cells (Fereshteh et al., 2012; Kotula et al., 2014), the interaction of β-arr1 with the DNA-binding Enhancer of Zeste Homologue 2 (EZH2) protein mediates BCR/ABL histone acetylation during tumor progression (Qin et al., 2014). In addition, it has been proved that the self-renewal ability of the leukemia initiating cell-enriched subpopulation is linked to the ability of β-arr1 to promote the activity of the DNA methyltransferase 1 on PTEN promoter region, thus reducing the expression of PTEN (Shu et al., 2015). At the same time, nuclear β-arr1 represses the senescence of leukemic cells by interaction with hTERT, thus enhancing telomerase activity and telomere length (Liu et al., 2017), providing novel insights into the β-arr1-mediated regulation of leukemic cells. In Sonic Hedgehog medulloblastoma, where aberrant Sonic Hedgehog/Gli (Hh/Gli) signaling pathway is a critical regulator of tumor initiation and progression, β-arr1 promotes p300-mediated acetylation of Gli1 inhibiting its function, acting as negative regulators of self-renewal (Miele et al., 2017). All these findings, and in particular the specific contributions of β-arr1 for acetylation/methylation mechanisms or interactions with transcriptional factors or regulators, establish a new paradigm in multimodality of β-arr1 in controlling gene expression in cancer. However, their integration will have to be complemented with other studies in specific tumors and cell types, occurring during tumor development and metastasis. Future research will need to address whether similar mechanisms might occur for other GPCRs and open new ways to understand new nuclear interactions of β-arr1 in cancer and to obtain the effective knowledge of how β-arr1 is complicit in the epigenetic control of cancer progression.

## Role of β-arr2 in Cancer Progression

Although β-arr1 and β-arr2 show high degree of sequence and structural similarity and functional overlap (Srivastava et al., 2015), emerging evidences establish an involvement of β-arr2 in cancer growth and progression, with contradictory results. Previous studies demonstrated that β-arr2 depletion promoted tumor growth and angiogenesis in a murine model of lung cancer (Raghuwanshi et al., 2008) and that low expression of β-arr2 is significantly associated with aggressive pathologic features and is predictive of poor patient prognosis, as observed in lung and hepatocellular carcinoma (Sun et al., 2016; Cong et al., 2017). In prostate cancer, β-arr2 inhibits cell viability and proliferation by downregulation of FOXO1 and represses AR signaling, and AR expression/activity negatively correlates with β-arr2 expression (Lakshmikanthan et al., 2009; Duan et al., 2015). By contrast, other results are consistent with the idea that β-arr2 action provides a supportive role in the development of human tumors, and β-arr2 is overexpressed in different human tumors, including breast and renal cell carcinoma, correlating with advanced stage and decreased patient survival, and mediates different tumor-promoting effects, such as cell migration and invasion (Sun et al., 2002; Ge et al., 2004; Alemayehu et al., 2013; Masannat et al., 2018). In both myeloid leukemia and ovarian cancer cells, the cross-signaling between β-arr2 and Wnt controls cell proliferation and metastasis through the interaction with c-Src followed by EGFR transactivation (Luttrell et al., 1999; Rosanò et al., 2009). The interaction of β-arr2 with c-Src is also implicated in regulating cell cycle progression and metastatic tumor growth in mice and further expanding the role of β-arr2 (Zhang et al., 2011). Very recently, a new role of β-arr2 has been linked to Hh signaling and medulloblastoma tumorigenesis, controlling SuFu-Gli3 complex, as a major control node in Hh signaling. In particular, it has been demonstrated that in the absence of Hh signaling the interaction of β-arr2 with the E3 ligase Itch and Suppressor of Fused (SuFu), a tumour suppressor gene, promotes the processing of Gli3 transcription factor into a cleaved repressor form, GLI3R, unveiling a new role of β-arr2 in controlling the immunosuppressive function of SuFu and maintaining the signaling off in the absence of ligand (Infante et al., 2018). These results further point out that function specialization of β-arr isoforms might exist in cancer, implying that different roles of β-arr2 function may be cell context- and cancer type-dependent, and that many other studies are needed to fully understand the mechanisms underlying the role of β-arr2 in cancer.

## Future Exploration of Targeting Convergent GPCR/β-arr-Dependent Mechanisms in Cancer

The role of β-arr-mediated network in executing the functional consequences of GPCR signaling in tumor cells is providing new insight into the mechanisms that underlie cell migration and the acquisition of invasive traits. Besides the complexity of GRK-mediated signaling that could allow compensatory networks to strengthen cancer progression suggesting the feasibility of therapeutic strategies using GRK inhibitors (Nogués et al., 2017, 2018), the central theme that has emerged from this review is the critical role of GPCR/β-arr-driven signaling in rewiring the complex signaling network sustaining cancer progression. This indicates that targeting GPCR/β-arr pathways might represent a new route of therapeutic intervention by developing precision medicines with tailored efficacy profiles for cancer-specific context. The existence of biased ligands that can transduce intracellular signaling from a GPCR by favoring either the G-protein or the β-arr-mediated signaling pathways strongly leads to the development of signal-biased drugs (Smith et al., 2018; Xiao and Sun, 2018). In this context, findings obtained by specifically targeting ET-1R/β-arr1-driven pathways by using small molecule ET-1R antagonist demonstrated a potential therapeutic approach for controlling metastatic progression (Rosanò et al., 2014; Cianfrocca et al., 2016, 2017; Semprucci et al., 2016; Chellini et al., 2018; Di Modugno et al., 2018). In addition to directly targeting GPCRs, emerging data suggest the possibility to selective disruption of β-arr interactions by inhibiting the linked functional responses, as an RNA aptamer targeting βarr2 and a synthetic intrabody fragment recognizing βarr, or by specifically interfering with GPCR/β-arr interaction, revealing new ways of therapeutically targeting β-arr-driven convergent signals in patients to hamper metastatic dissemination (Chaturvedi et al., 2018).

## Conclusions

Recent technological advancements in structural and cell biology have provided crucial insights into the molecular mechanisms of GPCR signaling mediated by both β-arrs and G-proteins, shedding additional light on the dynamic assembly and disassembly of GPCR signaling complexes (Alvarez-Curto et al., 2016; O’Hayre et al., 2017; Eichel et al., 2018; Grundmann et al., 2018; Gurevich and Gurevich, 2018; Gutkind and Kostenis, 2018; Latorraca et al., 2018; Luttrell et al., 2018). In these new advancements, studies limited to ERK signaling by using genome editing to modulate G protein or β-arr expression and/or function suggest that β-arrs, rather than being active GPCR transducers, are critical initiator of G-protein-mediating signaling cascade, acting as “rheostat” (Gutkind and Kostenis, 2018), uncovering new role of β-arr as dictating factors of G-protein-dependent signaling activation.

Although aspects of the GPCR/β-arr signaling network had been established previously, the novelty of the recent studies highlighted in this review is the ability of β-arr to orchestrate a complex signaling network that specifically controls in a fine-tuning manner, the time, intensity, and space of GPCR-mediated signaling flow to regulate distinct steps of tumor invasion, extravasation, and metastatic spread. Looking forward, a better understanding of how the different types of GPCRs contribute to β-arr-driven signaling activation in tumor cells and by tumor microenvironment and their interaction with other surface receptors is needed. These studies should consider the role of mechanical forces imposed by the ECM and tissue microenvironment in GPCR/β-arr-mediated actin cytoskeleton remodeling and should attempt to delineate the specific contributions of different effectors (such as Rho GTPase family members) to promote cytoskeleton effects related to cell motility and invasiveness in the context of GPCR/β-arr signaling (Figure 1). However, remains to be learned both about how β-arr-1 mediates gene expression changes to execute the GPCR-induced pro-metastatic effects in tumor cells and about how β-arr-1/-2 and G-protein-mediated effects may differ in this regard. The possibility that cytoplasmic and nuclear β-arrs are regulated by several cues and contribute to unexplored relevant aspects of tumor progression should also be considered. The impact of this work is likely to be substantial, given the intense interest in targeting GPCR/β-arr signaling as a therapeutic approach to inhibiting metastatic progression in cancer patients and highlighting the need for translation of preclinical insights into clinical applications. Considering the role of GPCR/β-arr-driven signaling in cancer progression and many efforts in using GPCR antagonists to dampen specifically β-arr-dependent signaling, other studies are needed not only to rewire the complexities of β-arr signaling networks and the functional effects in cancer but also to strongly improve therapeutic targeting of GPCR in cancer. In this regard, additional studies using adequate patient-derived models to further analyze the blockade of potential β-arr-dependent signaling machinery are needed to instruct treatment options in the clinic.

## Author Contributions

Both authors listed have made a substantial, direct and intellectual contribution to the work, and approved it for publication.

### Conflict of Interest Statement

The authors declare that the research was conducted in the absence of any commercial or financial relationships that could be construed as a potential conflict of interest.
